# Case Report: Male Lobular Breast Cancer in Hereditary Cancer Syndromes

**DOI:** 10.3389/fonc.2022.891426

**Published:** 2022-05-24

**Authors:** Ileana Carnevali, Gianluca Tedaldi, Valeria Pensotti, Nora Sahnane, Donata Micello, Francesca Rovera, Fausto Sessa, Maria Grazia Tibiletti

**Affiliations:** ^1^ Unit of Pathology, Ospedale di Circolo, Azienda Socio Sanitaria Territoriale (ASST)-Sette Laghi, Varese, Italy; ^2^ Research Center for the Study of Hereditary and Familial Tumors, Department of Medicine and Surgery, University of Insubria, Varese, Italy; ^3^ Biosciences Laboratory, Istituto Ricerca e Cura a Carattere Scientifico (IRCCS) Istituto Romagnolo per lo Studio dei Tumori “Dino Amadori” – Istituto Romagnolo per lo Studio dei Tumori (IRST) S.r.l., Meldola, Italy; ^4^ Cogentech s.r.l. Società Benefit a Socio Unico, Milan, Italy; ^5^ Inter-Hospital Pathology Division, Istituto di Ricovero e Cura a Carattere Scientifico (IRCCS) MultiMedica, Milan, Italy; ^6^ Department of Medicine and Surgery, University of Insubria, Varese, Italy

**Keywords:** male lobular breast cancer, inherited cancer syndromes, BRCA, CDH1, case report

## Abstract

**Background:**

Lobular breast carcinoma (LBC) is considered an exceptionally rare disease in men, including only 1% of all male breast malignancies. The majority of LBCs have negative immunohistochemical staining for E-cadherin (*CDH1*) expression, and the loss of *CDH1* function was traditionally implicated in the tumorigenesis of diffuse gastric cancer as well as LBC. It is well recognized that LBC in women could be involved in both hereditary breast and ovarian cancer (HBOC) and hereditary diffuse gastric cancer (HDGC) syndromes; however, there are no data present in literature about the involvement of male LBC in these inherited conditions.

**Methods:**

*BRCA1*, *BRCA2*, and *CDH1* genes were performed on DNA from peripheral blood using next-generation sequencing (NGS), Sanger sequencing, and multiplex ligation-dependent probe amplification analyses. *BRCA2* and *CDH1* somatic gene analyses were performed on breast tumoral DNA using the NGS sequencing approach.

**Results and conclusions:**

Here, we describe two men affected by LBC, the carriers of a pathogenic variant of *BRCA2* and *CDH1* genes, respectively. Our data, including somatic and germline results, demonstrate a strong relationship between male LBC and HBOC/HDGC syndromes, excluding a sporadic origin of LBC in these two patients. Male LBC could represent a sentinel cancer for inherited syndrome identification, and early identification of cancer susceptibility could improve cancer prevention both for men and women in these families. The history of the LBC patient carrier of the *CDH1* variant suggests to include male LBC genetic testing criteria and male breast surveillance in HDGC guidelines.

## Introduction

Male breast cancer (MBC) is a rare entity, representing 1% of all breast, male and female, cancers. Invasive lobular breast carcinoma (LBC) is exceptionally rare in men, comprising only 1% of all male breast malignancies (1), probably because lobular development does not occur in the male breast. The origin of the lobular histological type of breast carcinoma in men remains largely unexplained and, due to its extremely uncommon occurrence, knowledge on the natural history of disease progression, clinical presentation, treatment management, and prognosis in men is, to date, very limited (2).

Invasive LBC is more likely to be estrogen and progesterone positive compared with invasive ductal carcinoma, and it is usually HER-2, p53, and EGFR negative. The majority of LBCs have negative immunohistochemical staining for E-cadherin expression, which is the main regulator of the lobular phenotype in breast cancer. E-cadherin dysfunction is responsible for the discohesive histomorphological characteristics of LBC. The loss of *CDH1* function was traditionally implicated in the tumorigenesis of diffuse gastric cancer (DGC), and both LBC and DGC share the same histopathological characteristics, including individual or small clusters of discohesive cell growth pattern.

It is well known that a subset of MBCs is due to inherited conditions; in particular, *BRCA1* and *BRCA2* are the most involved genes. The genetic susceptibility linked to these genes contributes to the pathogenesis of many MBCs (3); however, the relationship between the MBC lobular histotype and cancer susceptibility is so far unknown.

A family history of breast and ovarian cancers is reported in approximately 15%–20% of MBCs, and approximately 10% of MBC patients carry *BRCA2* variants, while few patients carry the pathogenic variants of *BRCA1* (4).

Very recently, Rizzolo et al., in a large Italian study, identified MBC patients with the germline pathogenic variants of other genes including *PTEN*, *TP53*, *PALB2*, and *CHEK2* (5), suggesting that MBC susceptibility could involve additional genes other than *BRCA1/2*.

Here, we describe two male patients affected by LBC, the carriers of *BRCA2* and *CDH1* germline pathogenic variants, respectively. These two cases suggest that male LBC could be associated with inherited cancer conditions.

## Methods

### Somatic Analysis

#### Immunohistochemistry (IHC) Analysis

Formalin-fixed, paraffin-embedded (FFPE) tumor sections were completely processed automatically on a VENTANA BenchMark ULTRA immunostainer using ultraView universal DAB detection Kit (Ventana Indianapolis, Indiana), as routinely. According to the suggested protocols, after antigen retrieval with a Cell Conditioning 1 solution, the sections were incubated with the following primary antibodies: anti-ER (clone SP1, Ventana), anti-PR (clone 1E2, Ventana), anti-HER2 (clone 4B5, Ventana), anti-p53 (clone DO7, Ventana), and anti-Ki67 (clone MIB1 1/100, DAKO).

##### DNA Extraction From Formalin-Fixed Paraffin-Embedded Tissue

Tumoral DNA was obtained after the macrodissection of FFPE samples with 80% of neoplastic cells, as previously described by Carnevali etal. (6).

##### Next-Generation Sequencing

For the analysis of *BRCA2* somatic variants on patient 1, the NGS library was prepared with SOPHiA homologous recombination solution (SOPHiA GENETICS), starting from 80 ng of DNA extracted from FFPE tumor tissue, and was run on the Miseq System (Illumina). Results were analyzed with SOPHiA DDM platform v. 2.6.3 (SOPHiA GENETICS).

For the analysis of *CDH1* somatic variants on patient 2, the NGS library was prepared with Nextera Flex for Enrichment (Illumina), starting from 100 ng of DNA extracted from FFPE tumor tissue, and enriched for a custom panel (Integrated DNA Technologies), including the *CDH1* gene and other cancer predisposition genes. The enriched library was run on the Miseq System (Illumina), and results were analyzed with Miseq Reporter Software v. 2.6.2 (Illumina).

#### Methylation Analysis

*CDH1* methylation was assessed, addressing six CpG sites (exact position: GrCh37/Hg19 chromosome 1668771035, 68771037, 68771045, 68771051, 68771059, 68771064, 68771073) in the gene promoter by pyrosequencing on bisulfite-converted DNA. About 200ng of DNA was bisulfite-treated by using EZ DNA Methylation Kit (Zymo Research, Irvine, CA, USA) and then amplified using TaKaRa EpiTaq™ HS reagents (Takara Bio Inc, Shiga, Japan). Primers used for PCR reactions were the following: Forward 5’-AGTAATTTTAGGTTAGAGGGTTA, Reverse 5’-Biotin- ACCACAACCAATCAACAAC while sequencing primer was 5’- ATTTTAGGTTAGAGGGTTAT. The cut-off value to call the presence of methylation was 10%.

### Germline Analysis

#### DNA Extraction

Whole blood DNA was isolated through the MagCore^®^ Super automatic workstation with the MagCore^®^ Genomic DNA Whole Blood Kit (Diatech LabLine SRL, Jesi, Italy).

#### Sanger Sequencing

*CDH1* coding exons (1-16) and flanking regions were simultaneously amplified at the annealing temperature of 60°C, with the AmpliTaq Gold kit (Applied Biosystems; Thermo Fisher Scientific, Inc., Waltham, MA, USA). Sequencing was performed on purified PCR products by using BigDye^®^ Terminator v.3.1 Cycle Sequencing kit (Thermo Fisher Scientific, Inc.) and run on the 3500 Dx Genetic Analyzer (Life Technologies) after purification with Agencourt CleanSeq^®^-Beckman Coulter. Sequences were analyzed by Mutation Surveyor^®^ Software v5.1.0 (SoftGenetics, LLC, State College, PA, USA). The same method was applied to confirm the *BRCA2* mutation identified in case 2 by NGS. Primers are available on request.

#### Multiplex Ligation-Dependent Probe Amplification

The analysis of large deletions and duplications of *CDH1*, *BRCA1*, and *BRCA2* was carried out on the DNA extracted from peripheral blood with the CDH1 SALSA MLPA KIT - P083 (C2), BRCA1 SALSA MLPA KIT - P002 (D1), BRCA2 SALSA MLPA KIT - P045 (C1) probemixes (MRC-Holland, Amsterdam, Netherlands), following manufacturer’s instructions. MLPA products were run on the 3730Xl DNA Analyzer (Applied Biosystems; Thermo Fisher Scientific, Inc., Waltham, Massachusetts, United States) with the Gene Mapper Module (Applied Biosystems; Thermo Fisher Scientific, Inc.). Results were analyzed through the Gene Marker Software v2.7.0 (SoftGenetics, LLC, State College, PA, USA).

#### Next-Generation Sequencing

Approximately 10–25 ng of dsDNA, according to Qubit dsDNA HS assay kits fluorometric quantification (Thermo Fisher Scientific Inc.), were amplified in multiplex reactions, spanning the entire coding region of the *BRCA1* and *BRCA2* genes, including flanking intronic sequences (+/-20bp). The NGS library was created using TruSeq Custom Amplicon v.1.2 (TSCA) technology (Illumina Inc., San Diego, CA, USA), and sequencing was performed on MiSeq (Illumina Inc.), using a 2 × 150 bp paired-end module. Data collection was done with the MiSeq Reporter (MSR) software v.2.6.2.3. The run quality was evaluated by Illumina Sequencing Analysis Viewer v.1.9.1, while the annotation of the vcf files was performed with a customized bioinformatics pipeline. Paired-end reads were mapped on the Human hg19 genome. A full coverage of the coding regions was obtained, with a minimum read depth of 50X and an average read depth of 3,500X/sample.

#### Variant Classification

The identified genetic variants were divided into five classes according to the International Agency for Research on Cancer (IARC) recommendations (7) and classified in accordance with the guidelines of the American College of Medical Genetics (ACMG) (8) and the most recent guidelines on *CDH1* variant classification (9). The *BRCA2* variant was also classified according to the ENIGMA Consortium guidelines (10), obtaining the same class of pathogenicity.

## Results

### Patient 1

We herein described the case of a 63-year-old man showing unilateral gynecomastia in the right breast and an underlying palpable nodule. Ultrasound examination showed an irregular right periareolar hypoechogenic nodular formation of 23 mm with suspected posterior extension. In the homolateral axillary site, a possible adenopathy of approximately 10 mm was visible. In December 2016, he underwent right mastectomy; sentinel lymph node biopsy intraoperative examination revealed metastatic cancer cells and, subsequently, complete axillary lymphadenectomy.

The final diagnosis, from the pathology examination, was LBC with a solid growth pattern, poorly differentiated. In addition, LBC showed perineoplastic vascular invasion; the nipple had focal infiltration and the areolar skin was affected by neoplastic infiltration up to the reticular dermis. The resection margins were free from neoplasia. Thirty axillary lymph nodes were examined, and none showed signs of metastasis.

**Figure 1:**
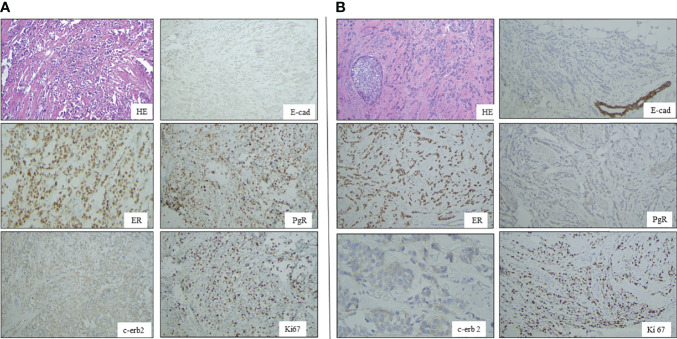
Panel **A** shows Hematoxilin-Eosin (HE) picture and immunohystochemical results of E-cadherin (B), estrogen receptor (D), progesterone receptor (C), HER2 and Ki67 expressions in breast cancer of case 1 Panel **B**: A shows Hematoxilin-Eosin (HE) picture and immunohystochemical results of E-cadherin (B), estrogen receptor (D), progesterone receptor (C), HER2 and Ki67 expressions in breast cancer of case 2.

The IHC analysis of ER, PgR, Her2/neu, MIB1, p53, and E-cadherin proteins on FFPE tissue sections demonstrated immunoreactivity for ER and PgR (90% and 75%, respectively) and the loss of p53 e E-cadherin expression (**Figure 1**). The proliferation index (MIB-1) was detected in 32% of neoplastic cells, and Her2/neu was evaluated as a 1+ score (ASCO guidelines 2018). TNM staging was pT1cN1a.

After a multidisciplinary discussion, the patient underwent surgery and 4 cycles of adjuvant chemotherapy with anthracyclines, followed by 12 cycles q1 of paclitaxel. After a whole chemotherapy course, the patient underwent fractioned radiation therapy with a total dose of 50 and 66 Gy applied to the chest wall and to the surgical site, respectively. After radiotherapy, the patient began a 5-year treatment with tamoxifen; he is still alive in a follow-up after 5 years from diagnosis.

#### Family History and Germline Analysis

**Figure 2:**
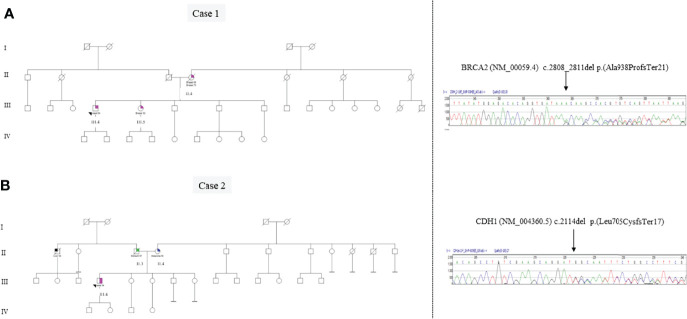
Panel **A**: Genetic pedigree of case 1 and Sanger sequencing electropherogram of the BRCA2 exon 11 variant c.2808_2811delACAA (p.Ala938ProfsTer21). The alignment to the reference sequence of BRCA2 NM_000059.3 was created by the Mutation Surveyor® Software (v5.1.0). Panel **B**: Genetic pedigree of case 2 and Sanger sequencing electropherogram of the CDH1 exon 13 variant c.2114delT (p.Leu705CysfsTer17). The alignment to the reference sequence of CDH1 NM_004360.3 was created by the Mutation Surveyor® Software (v5.1.0).

Case 1 (III.4 in a pedigree **Figure 2A**) was referred in 2016 to our Genetic Counseling Service, ASST Sette Laghi Varese, during his follow-up. As shown in **Figure 2**, the proband’s mother (II.2) died at age 85 after the diagnosis of bilateral breast cancer at ages 60 and 70, and the proband’s sister (III.2) developed breast cancer at age 60. There is no other report of malignancy in this family that, in agreement with FONCAM and NCCN guidelines (11, 12), was suspected of HBOC syndrome (ORPHA 145).

The genetic analysis of *BRCA1* and *BRCA2* genes in this patient revealed the germline pathogenic variant c.2808_2811delACAA p.(Ala938ProfsTer21) in heterozygosity (VAF ~0.5) in the *BRCA2* gene (transcript NM_00059.4) using NGS and subsequently confirmed by Sanger sequencing.

The *CDH1* gene was also analyzed using both Sanger Sequencing and MLPA approaches, and no variants were identified. The *BRCA2* pathogenic variant results in a premature termination codon predicted to cause a truncated or absent BRCA2 protein due to nonsense-mediated decay, which is a commonly known mechanism for disease. It is seen in the general European population at a low overall frequency of 0.0018%, as reported in the Genome Aggregation Database (13). This variant is known to be one of the most common pathogenic variants in non-Ashkenazi Caucasians (14–16), and it has been reported in association with familial and early-onset breast and/or ovarian cancers (17–19) and also in individuals with prostate cancer (20, 21).

The *BRCA2* pathogenic variant was also investigated in the patient’s two sons (IV.8, IV.9), who inherited the same *BRCA2* pathogenic variant. No other members of this family decided to perform genetic testing.

#### Tumor Analysis

The molecular analysis of the *BRCA2* gene on the tumoral DNA of case 1 revealed the presence of 8 variants, 7 of which were also present in the germline DNA, previously identified as germline variants from blood (**Table 1**). The software detected in the tumor tissue the *BRCA2* variant c.865A>C p.(Asn289His) (rs766173) not present in the germline DNA with a variant allele frequency (VAF) of 0.013.

**Table 1:** *BRCA2* somatic variants of the patient 1.

Gene	Location	Chromosome	Genomic position (hg19)	complementary DNA (cDNA) (NM_00059)	Protein (NP_000050)	VAF	Depth	The Single Nucleotide Polymorphism Database (dbSNP)	ClinVar	Varsome
*BRCA2*	exon 10	chr13	32906480	c.865A>C	p.(Asn289His)	0.013	2001	rs766173	Benign	Benign
*BRCA2*	exon 11	chr13	32911300	c.2808_2811del	p.(Ala938ProfsTer21)	0.75	1975	rs80359351	Pathogenic	Pathogenic
*BRCA2*	exon 11	chr13	32911888	c.3396A>G	p.(Lys1132=)	0.18	2672	rs1801406	Benign	Benign
*BRCA2*	exon 11	chr13	32912560	c.4068G>A	p.(Leu1356=)	0.23	2530	rs28897724	Benign	Benign
*BRCA2*	exon 11	chr13	32913055	c.4563A>G	p.(Leu1521=)	0.99	3924	rs206075	Benign	Benign
*BRCA2*	exon 11	chr13	32915005	c.6513G>C	p.(Val2171=)	1.00	2821	rs206076	Benign	Benign
*BRCA2*	exon 14	chr13	32929387	c.7397T>C	p.(Val2466Ala)	1.00	1918	rs169547	Benign	Benign
*BRCA2*	intron 16	chr13	32936646	c.7806-14T>C	–	0.76	1662	rs9534262	Benign	Benign

VAF, variant allele frequency.

Three of the 7 *BRCA2* variants, present both in the germline and tumoral DNA (rs206075, rs206076, rs169547), were in homozygosity (VAF~1.00). The other 4 variants (rs80359351, rs1801406, rs28897724, rs9534262) were in heterozygosity in the germline DNA (VAF~0.5) but respectively showed the VAFs of 0.75, 0.18, 0.23 and 0.76 in the tumoral DNA, indicating an allelic imbalance (AI) and suggesting the presence of a loss of heterozygosity (LOH).

### Patient 2

In 2017, a 34-year-old Caucasian man had a diagnosis of right breast nodule in the peri-retroareolar site. Breast ultrasound showed a pseudo-nodular formation with poorly defined boundaries. The subsequent mammography showed a small parenchyma, while positron emission tomography (PET) analysis was negative. The patient underwent a right mastectomy with axillary dissection after the evaluation of a sentinel lymph node that was positive with 1 cm of the metastatic localization. The neoplasm had a maximum diameter of 1 cm, hard consistency, and irregular margins.

The histopathological analysis revealed a lobular invasive classical type (Indian file carcinoma) (Breast Tumours WHO Classification of Tumours, 5th Edition, Volume 2**))**, G2 infiltrating the perineural spaces, the skin derma of the areola, and nipple. No metastasis was observed among the 21 examined lymph nodes of the right axilla. The TNM staging of this tumor was pT1b pN1a G2.

Immunohistochemistry was done for ER, PgR, Her2/neu, MIB1, and p53 on FFPE sections, and positive immunoreactivity for ER and PgR (98% and 10%, respectively) was observed.

The proliferation index (MIB-1) revealed 40% immunoreactivity, and Her2/neu was evaluated as a 1+ score (ASCO guidelines 2013). Immunohistochemistry showed negativity for E-cadherin and p53 proteins (**Figure 1**).

The patient underwent 4 cycles of AC + 12 weekly paclitaxel chemotherapy cycles followed by long- term hormonotherapy with tamoxifen. Clinical follow-up was performed every year. During 4 years of follow-up, radiological follow-up through mammography or ultrasound, no signs of recurrence were observed.

#### Family History and Germline Analyses

Case 2 was referred to the Genetic Counseling Service of the Department of Pathology at Varese Hospital in 2018 after his LBC diagnosis.

As can be seen in the pedigree (**Figure 2B**), the patient’s father died at the age of 57 due to a DGC. His paternal uncle died at 58 years of age due to hepatocellular carcinoma without histological diagnosis, and the proband’s mother (II.4) was affected by melanoma at 50 years.

In case 2, the *BRCA1* and *BRCA2* genes were both analyzed by NGS, to identify single-nucleotide variations and small ins/del, and by MLPA to identify extended deletions/duplications non-detectable by sequencing. The *CDH1* gene was also analyzed using both Sanger Sequencing and MLPA approaches. Germline analysis in the proband revealed the pathogenic variant c.2114delT p.(Leu705CysfsTer17) in the heterozygosity (VAF~0.5) *CDH1* gene (transcript NM_004360.5), leading to the diagnosis of hereditary diffuse gastric cancer (HDGC) syndrome (ORPHA 26610) (**Figure 2**), while *BRCA1/2* analysis did not reveal any variant. The pathogenic *CDH1* variant results in a premature termination codon predicted to cause a truncated or absent E-cadherin protein due to nonsense-mediated decay. The identified pathogenic *CDH1* variant is not present in population databases but has been already described in a DGC patient (22). Unfortunately, no other members of this family decided to perform *CDH1* genetic testing for cancer prevention. Patient 2 was sent to a reference center for high-risk stomach surveillance for DGC, as suggested by international guidelines (Blair et al.).

#### Tumor Analysis

The analysis of somatic variants on case 2 revealed the presence of 7 variants in the *CDH1* gene (**Table 2**). Two variants (rs3743674, rs1801552) were in homozygosity (VAF~1.00). The other 5 variants (rs16260, rs45625236, rs2276330, c.2114delT, rs1801026) showed VAFs of 0.17, 0.75, 0.76, 0.67, and 0.68, respectively, indicating an allelic imbalance (AI) and suggesting the presence of an LOH. The methylation analysis of the *CDH1* promoter was negative, suggesting that the methylation mechanism is not involved in the carcinogenesis of this LBC.

**Table 2:** *CDH1* somatic variants of the patient 2.

Gene	Location	Chromosome	Genomic position (hg19)	complementary DNA (cDNA) (NM_004360)	Protein (NP_004351)	VAF	Depth	The Single Nucleotide Polymorphism Database (dbSNP)	ClinVar	Varsome
*CDH1*	upstream	chr16	68771034	c.-285C>A	–	0.17	1098	rs16260	Benign	Benign
*CDH1*	intron 1	chr16	68771372	c.48+6C>T	–	1.00	531	rs3743674	Benign	Benign
*CDH1*	intron 1	chr16	68771419	c.48+62_48+63insCGTGCCCCAGCCC	–	0.75	700	rs45625236	–	Benign
*CDH1*	intron 12	chr16	68857289	c.1937-13T>C	–	0.76	1267	rs2276330	Benign	Benign
*CDH1*	exon 13	chr16	68857441	2076T>C	p.(Ala692=)	1.00	1847	rs1801552	Benign	Benign
*CDH1*	exon 13	chr16	68857479	c.2114del	p.(Leu705CysfsTer17)	0.67	1665	–	–	Pathogenic
*CDH1*	3’UTR	chr16	68867456	c.*54C>T	–	0.68	464	rs1801026	Benign	Benign

## Discussion

LBC is a distinct type of breast carcinoma characterized by its histopathological features of discohesive cells, representing 5%–15% of invasive breast carcinomas in women. The occurrence of LBC is exceptionally rare in men (16). In our Breast Unit, MBCs were systematically investigated for inherited conditions since 2008–2018 and 37 affected men were referred to Cancer Genetic Counselling.

Genetic analysis revealed an inherited condition involving MBC in 10 out of 37 (27%) cases, including 7 carriers of *BRCA1/2* germline pathogenic variants (3 *BRCA1* and 4 *BRCA2*) and 3 carriers of pathogenic variants of other genes including *APC*, *MUTYH*, and *CDH1*. Invasive ductal carcinomas were diagnosed in 35 out of 37 MBCs including sporadic and inherited cases. Only two MBC patients with a lobular histological type (2 out of 37) described here resulted to have an inherited cancer syndrome.

It is well recognized that LBC in women could be involved in both HBOC (ORPHA 145) and HDGC syndromes (ORPHA 26610) (23, 24); however, no data are present in literature about the involvement of male LBC in these inherited conditions.

Here, we described two men who are affected by LBC and carriers of *BRCA2* and *CDH1* pathogenic variants, respectively. Both of these patients are affected by two distinct inherited syndromes involving LBC, and, to our knowledge, this is the first report demonstrating the strong relationship between an inherited condition and LBC carcinogenesis in men.

Genetic analyses performed on the tumoral DNA of both patients suggested that, in case 1, male LBC arose from *BRCA2* inactivation due to the presence of a germline pathogenic variant in the gene, followed by a possible somatic loss of the second allele (LOH) of the gene. Similarly, in case 2, LBC seems due to both the germline pathogenic variant of the *CDH1* gene, followed by a possible loss of the second allele in tumoral DNA, which represents the second hit of *CDH1* tumor suppressor gene inactivation process. Interestingly, this *CDH1*-mediated carcinogenesis correlated with a lobular morphological feature in an MBC.

The identification of inherited cancer syndromes through the genetic analysis of the two LBC male patients prompted us to offer a cascade genetic testing in both of these two families, in order to improve cancer prevention using dedicated breast and ovarian cancer surveillance protocol in the family members who are a carrier of the *BRCA2* pathogenic variant and in breast and gastric cancer patients’ family members who are a carrier of the *CDH1* pathogenic variant in agreement with the prevention guidelines of these syndromes (11, 12, 24). It is noteworthy that the endoscopic surveillance performed in patient 2 allowed to identify an early DGC.

In summary, our data including somatic and germline genetic results demonstrate a strong relationship between male LBC and *BRCA2* and *CDH1* genes, excluding a sporadic origin of LBC in these men.

Taking into account that male LBC is a very rare cancer, this condition could represent a sentinel cancer for inherited syndrome identification, and, furthermore, the early identification of inherited cancer syndromes could improve cancer prevention both for men and women in these families. In particular, the clinical and genetic history of the LBC patient carrier of the *CDH1* pathogenic variant suggests us to include male LBC in the genetic testing criteria and male breast surveillance in local and HDGC guidelines (23).

## Data Availability Statement

The data presented in the study are deposited in the Insubria Ethics Committee repository, approved in 2014 and revised in study number 147/2021. Further inquiries can be directed to the corresponding author/s.

## Ethics Statement

The studies involving human participants were reviewed and approved by Comitato Etico dell’Insubria. The patients/participants provided their written informed consent to participate in this study.

## Author Contributions

IC: genetic counseling, data management, and manuscript writing. GT: somatic DNA analyses. VP: germline analysis. FS and DM: histological diagnosis and case revision. NS: methylation analyses. FR: clinical management. MT: design of the study, data management, and manuscript writing. All the authors of this manuscript directly participated in the planning, execution, and analysis of the study.

## Conflict of Interest

Author VP was employed by Cogentech s.r.l. Società Benefit a Socio Unico.

The remaining authors declare that the research was conducted in the absence of any commercial or financial relationships that could be construed as a potential conflict of interest.

## Publisher’s Note

All claims expressed in this article are solely those of the authors and do not necessarily represent those of their affiliated organizations, or those of the publisher, the editors and the reviewers. Any product that may be evaluated in this article, or claim that may be made by its manufacturer, is not guaranteed or endorsed by the publisher.
